# Distinct HLA associations of LGI1 and CASPR2-antibody diseases

**DOI:** 10.1093/brain/awy109

**Published:** 2018-05-18

**Authors:** Sophie Binks, James Varley, Wanseon Lee, Mateusz Makuch, Katherine Elliott, Jeffrey M Gelfand, Saiju Jacob, M Isabel Leite, Paul Maddison, Mian Chen, Michael D Geschwind, Eleanor Grant, Arjune Sen, Patrick Waters, Mark McCormack, Gianpiero L Cavalleri, Martin Barnardo, Julian C Knight, Sarosh R Irani

**Affiliations:** 1 Oxford Autoimmune Neurology Group, Nuffield Department of Clinical Neurosciences, University of Oxford, Level 3, West Wing, John Radcliffe Hospital, Oxford, OX3 9DS, UK; 2 Wellcome Centre for Human Genetics, University of Oxford, Oxford, OX3 7BN, UK; 3 UCSF Department of Neurology, 675 Nelson Rising Lane, San Francisco, CA 94158, USA; 4 Centre for Rare Diseases and Queen Elizabeth Neuroscience Centre, University Hospitals Birmingham, UK; 5 Department of Neurology, Queen’s Medical Centre, Derby Road, Nottingham NG7 2UH, UK; 6 Transplant Immunology and Immunogenetics Laboratory, Oxford Transplant Centre, Churchill Hospital, Oxford, UK; 7 Department of Molecular and Cellular Therapeutics, the Royal College of Surgeons in Ireland, Dublin, Ireland

**Keywords:** human leucocyte antigen, leucine-rich glioma-inactivated 1, contactin-associated protein 2, voltage-gated potassium channel, major histocompatibility complex

## Abstract

The recent biochemical distinction between antibodies against leucine-rich, glioma-inactivated-1 (LGI1), contactin-associated protein-2 (CASPR2) and intracellular epitopes of voltage-gated potassium-channels (VGKCs) demands aetiological explanations. Given established associations between human leucocyte antigen (HLA) alleles and adverse drug reactions, and our clinical observation of frequent adverse drugs reactions in patients with LGI1 antibodies, we compared HLA alleles between healthy controls (*n* = 5553) and 111 Caucasian patients with VGKC-complex autoantibodies. In patients with LGI1 antibodies (*n* = 68), HLA-DRB1*07:01 was strongly represented [odds ratio = 27.6 (95% confidence interval 12.9–72.2), *P* = 4.1 × 10^−26^]. In contrast, patients with CASPR2 antibodies (*n* = 31) showed over-representation of HLA-DRB1*11:01 [odds ratio = 9.4 (95% confidence interval 4.6–19.3), *P* = 5.7 × 10^−6^]. Other allelic associations for patients with LGI1 antibodies reflected linkage, and significant haplotypic associations included HLA-DRB1*07:01-DQA1*02:01-DQB1*02:02, by comparison to DRB1*11:01-DQA1*05:01-DQB1*03:01 in CASPR2-antibody patients. Conditional analysis in LGI1-antibody patients resolved further independent class I and II associations. By comparison, patients with both LGI1 and CASPR2 antibodies (*n* = 3) carried yet another complement of HLA variants, and patients with intracellular VGKC antibodies (*n* = 9) lacked significant HLA associations. Within LGI1- or CASPR2-antibody patients, HLA associations did not correlate with clinical features. *In silico* predictions identified unique CASPR2- and LGI1-derived peptides potentially presented by the respective over-represented HLA molecules. These highly significant HLA associations dichotomize the underlying immunology in patients with LGI1 or CASPR2 antibodies, and inform T cell specificities and cellular interactions at disease initiation.

## Teaser

The mechanisms responsible for the generation of LGI1- or CASPR2-antibodies are unknown. Binks *et al.* describe two strikingly dichotomous HLA-associations, most significantly in HLA-DRB1*07:01 for LGI1, and HLA-DRB1*11:01 for CASPR2. Prediction of antigen-specific HLA-binding peptides generates testable hypotheses for T cell identification.

## Introduction

The discovery of autoantibodies against leucine-rich, glioma-inactivated 1 (LGI1), contactin-associated protein 2 (CASPR2) (Irani *et al.*, 2010; Lai *et al.*, 2010) and, more recently, intracellular epitopes of voltage-gated potassium channels (VGKCs) (Lang *et al.*, 2017), have redefined the immunology of the VGKC-complex (Thieben *et al.*, 2004; Vincent *et al.*, 2004). Patient stratification by these antigenic targets has shown that the ‘double-negative’ VGKC-complex antibodies, those without LGI1 or CASPR2 reactivities, are observed across all ages, in healthy controls and in a variety of syndromes, many of which are not immune-mediated (Graus and Gorman, 2016; van Sonderen *et al.*, 2016; Lang *et al.*, 2017). In contrast, patients with LGI1 or CASPR2 antibodies often have clinically-indistinguishable late-onset forms of limbic encephalitis and neuromyotonia with associated dysautonomia, sleep disturbances, pain and seizures (Irani *et al.*, 2010; Lai *et al.*, 2010; Klein *et al.*, 2013; Gadoth *et al.*, 2017). While these features occur at different rates in LGI1- versus CASPR2-antibody cohorts, only faciobrachial dystonic seizures (FBDS) robustly predict LGI1 reactivity (Irani *et al.*, 2011; Gadoth *et al.*, 2017; Thompson *et al.*, 2018). Furthermore, these two autoantibodies are both often of the IgG4 subclass and frequently co-exist in patients with the ultra-rare Morvan’s syndrome (Irani *et al.*, 2012; Ariño *et al.*, 2016). The striking overlaps of these rare neurological features and autoantibodies, and the frequent co-expression of their antigenic targets within mammalian CNS-membrane complexes (Irani *et al.*, 2010; Binks *et al.*, 2018), suggest they are involved in autoimmunization. Indeed, this has been reported in abattoir workers with autoantibodies against VGKC-complexes and, less so, CASPR2 (Meeusen *et al.*, 2012). The nature of the available complexes, antigen presentation mechanisms and the available T cell repertoires are likely to determine which antigen dominates the ensuing T–B cell response. If so, human leucocyte antigen (HLA) variants, intimately related to antigen presentation, may play critical roles in distinguishing the aetiology of these syndromes.

Previously, high rates of adverse drug reactions were observed in patients with LGI1 antibodies, typically secondary to antiepileptic drugs (AEDs) and, less so, corticosteroids (Irani *et al.*, 2011, 2013; Thompson *et al.*, 2018). As HLA variants have been implicated in several adverse drug reactions, including those associated with AEDs and immunosuppressants (McCormack *et al.*, 2011; Yip *et al.*, 2015), and have essential antigen-presenting functions (Trowsdale and Knight, 2013), we hypothesized that HLA associations existed in patients with LGI1 antibodies. Indeed, recently, HLA-DRB1*07:01, HLA-DQB1*02:02 and HLA-DRB4 were found to be present in varying proportions of patients with LGI1 antibodies in two cohorts totalling 40 patients, from Korea and the Netherlands (Kim *et al.*, 2017; van Sonderen *et al.*, 2017).

To extend these early observations, and given the hypothesis that the VGKC complex may be the initiating immunizing agent, we sought to compare and contrast HLA-associations in a sizeable cohort of clinically well-characterized patients with antibodies against LGI1, CASPR2, both LGI1 and CASPR2, and VGKCs, and *in silico* to identify peptides that may be presented by these HLA molecules.

## Materials and methods

### Patients

One hundred and eleven Caucasian patients were identified from previous studies (*n* = 51) (Irani *et al.*, 2011, 2013; Lang *et al.*, 2017), referrals to the Oxford Autoimmune Neurology Group (*n* = 46) or from the Autoimmune Encephalopathy Clinic, University of California San Francisco (*n* = 14). These patients had serum antibodies against LGI1 only (*n* = 68), CASPR2 only (*n* = 31), both LGI1 and CASPR2 (*n* = 3) or intracellular aspects of VGKCs (*n* = 9), as determined by previously described antigen-specific cell-based assays (Irani *et al.*, 2010; Lang *et al.*, 2017). Clinical phenotypes, including information relating to past medical history and adverse drug reactions (Table 1), were evaluated via direct patient and relative interviews and case-note reviews. All patients provided written informed consent (REC16/YH/0013 or the IRB 10-04905 approvals).

**Table 1:** Clinical features of patients with antibodies to VGKC complex proteins: LGI1, CASPR2, both LGI1 and CASPR2 or intracellular aspects of VGKCs

	LGI1	CASPR2	LGI1 and CASPR2	VGKC	LGI1 versus CASPR2 (*P-*value)*
Number of patients	68	31	3	9	ND
Median age at onset age (range)	63 (41–85)	68 (19–82)	56 (52–65)	43 (33–71)	ND
Female (%)	20 (29)	2 (6)	1 (33)	4 (44)	ND
**Clinical syndrome (%)**					
Epilepsy	8 (12)	2 (6)	0 (0)	4 (44)	ND
Encephalitis	58 (85)	18 (58)	1 (33)	2 (22)	ND
Morvan’s	1 (2)	3 (10)	2 (67)	0 (0)	ND
Isolated neuromyotonia	0 (0)	2 (6)	0 (0)	0 (0)	ND
Other^a^	1 (2)	6 (19)	0 (0)	3 (33)	ND
**Clinical features (%)**					
Any seizure	66 (97)	25 (81)	2 (67)	7 (78)	0.01
Faciobrachial dystonic seizures	47 (69)	0 (0)	1 (33)	0 (0)	<0.0001
Generalized seizure	26 (38)	10 (32)	2 (67)	6 (67)	NS
Amnesia	58 (85)	23 (74)	3 (100)	5 (56)	NS
Neuromyotonia	1 (2)	8 (26)	2 (67)	0 (0)	0.0003
Neuropathic pain	5 (7)	14 (45)	2 (67)	0 (0)	<0.0001
**Autoimmune features (%)**					
Other autoimmune disease^b^	19 (28)	7 (23)	0 (0)	0 (0)	NS
Atopy	8 (12)	2 (6)	2 (67)	1 (11)	NS
Adverse effects of corticosteroids^c^	32 (47)	5 (16)	0 (0)	1 (11)	0.004
Drug rash	24 (35)	1 (3)	0 (0)	0 (0)	0.0004
**Other features**					
Mean change in mRS (range)	1.6 (−3 to 4)	1.5 (0 to 4)	1.7 (0 to 3)	1 (0 to 2)	ND
Tumour (%)^d^	9 (13)	4 (13)	2 (67)	1 (11)	ND

Live cell-based assays were used for LGI1 and CASPR2 antibody determination (Irani *et al.*, 2010), and fixed assays to detect antibodies against the intracellular aspects of VGKCs (Lang *et al.*, 2017).

^a^Other diagnoses included movement disorders (*n* = 4, CASPR2, generalized chorea, hemifacial spasm, cervical dystonia and cerebellar ataxia), psychogenic amnesia (*n* = 2, VGKC antibodies), widespread non-neuropathic pain (*n* = 1, VGKC antibodies), axonal neuropathy (*n* = 1, CASPR2), psychosis (*n* = 1, CASPR2) and stroke (*n* = 1 with LGI1 antibodies). Two patients with antibodies against intracellular VGKC epitopes had epilepsy secondary to structural lesions.

^b^Autoimmune diseases in LGI1-antibody patients: [*n* = 19: diabetes (*n* = 1), heparin-induced thrombocytopaenia (*n* = 1), hyper- and hypothyroidism and Hashimoto’s thyroiditis (*n* = 8), multiple sclerosis (*n* = 1), myasthenia gravis (*n* = 1), neuromyelitis optica (*n* = 1), optic neuritis (*n* = 1), pernicious anaemia (*n* = 1), psoriasis (*n* = 6), Raynaud’s disease (*n* = 1), and ulcerative colitis (*n* = 1)] and CASPR2-antibody patients [*n* = 7: congenital adrenal hyperplasia, hypothyroidism, pernicious anaemia, pemphigus, polymyalgia rheumatica, psoriasis and Raynaud's disease (all *n* = 1)].

^c^Corticosteroid-related complications, sometimes multiple, in LGI1-antibody patients [*n* = 32: marked weight gain (*n* = 12), behavioural disturbance (*n* = 5) and diabetes (*n* = 5), or worsened diabetes (*n* = 1), insomnia (*n* = 4), fracture (*n* = 3), myopathy or muscle weakness (*n* = 3), skin thinning/easy bruising (*n* = 3), mania/hypomania (*n* = 2), poor wound healing or abscess (*n* = 2), ophthalmic infections (*n* = 2; keratitis and ophthalmic shingles), perforated abdominal viscus (*n* = 2), and one each of: avascular necrosis of the hip (AVN), cerebral venous sinus thrombosis, high INR and steroid-induced psychosis] and in CASPR2-antibody patients [*n* = 5: marked weight gain (*n* = 1), rash (*n* = 2), striae/thin skin/bruising (*n* = 2), and hallucinations (*n* = 1)].

^d^Tumours in LGI1-antibody patients (*n* = 9) were: basal cell carcinoma (*n* = 3), other skin – type not known (*n* = 2), bladder (*n* = 1), breast (*n* = 1), prostate (*n* = 1), dysplastic colonic polyp (*n* = 1) and in 4 CASPR2-antibody patients were: pancreatic (*n* = 1), prostate (*n* = 2), thymic cyst (*n* = 1).

NS = not significant; ND = not done; mRS = modified Rankin scale (as Thompson *et al.*, 2018).

*Statistical comparisons with Fisher’s exact test throughout.

### Genotyping, HLA imputation, verification and multi-locus haplotype-block construction

The Infinium Global Screening Array-24+v1.0 BeadChip with Illumina Infinium HTS custom markers were used for genotyping. We proceeded to impute HLA alleles using SNP2HLA at eight classical loci (HLA-A, HLA-B, HLA-C, HLA-DPA, HLA-DPB, HLA-DQA1, HLA-DQB1 and HLA-DRB1) at two-field resolution (Jia *et al.*, 2013; Neville *et al.*, 2017). To complement this, DRB1, DRB4 and DQ alleles underwent intermediate-resolution HLA-typing using PCR-sequence-specific primers (SSP), updated from Bunce *et al.* (1995). PCR-SSP defined the first-field plus a string of second-field possibilities: the highest frequency allele in Caucasians was considered most likely. For all discordant data, the PCR-SSP first-field was accepted as the final result. HLA alleles from 5553 Caucasian healthy controls (from Oxford Biobank) were available from imputation using the same platforms, and confirmed in 70 individuals within the same laboratory by PCR-SSP (Neville *et al.*, 2017). Probable haplotype blocks were calculated on the basis of a Bayesian algorithm using PHASE V2 software with 10 000 iterations for three haplotype blocks: HLA-C-B, HLA-DRB1-DQA1-DQB1, and HLA-DPA1-DPB1 (Stephens *et al.*, 2001; Stephens and Donnelly, 2003). Further details on genotyping and imputation are provided in the Supplementary material.

### Statistical analyses

For each antibody group, Fisher’s exact test (two-tailed) was used to compare the HLA allele and haplotype carrier frequencies between patients and the healthy control dataset. Hochberg’s method was used to correct for multiple comparisons. Corrected *P*-values < 0.05 were considered significant, and are presented. Odds ratios (ORs) were calculated using the median-unbiased estimation method.

### HLA binding predictions

The NetMHCIIpan 3.1 server model based on artificial neural networks (Andreatta *et al.*, 2015) evaluated HLA haplotype binding affinities for 15-amino acid-long consecutive overlapping peptides from full-length LGI1 and CASPR2 sequences (UniProt accession numbers O95970 and Q9UHC6, respectively). Predicted peptide affinities (nM) were compared to 200 000 random peptides of the same length to generate rank values: this measure is less susceptible to the intrinsic capacity of some HLA alleles to generate high-affinity predictions, and rank values (%) <3 were considered strong binders. As expected, consecutive 15-mer peptides with high rank values often shared a core sequence.

## Results

### Clinical differences between patients stratified by VGKC-complex autoantibody targets

Table 1 summarizes the clinical features of the 111 patients, subgrouped by their autoantibody specificities. In agreement with previous studies, onset ages were typically around 60 years, and patients with LGI1 or CASPR2 antibodies most frequently had encephalitis or epilepsy. FBDS were exclusive to patients with LGI1 antibodies (*P* < 0.0001) who had more seizures (*P* = 0.01) than patients with CASPR2 antibodies, where peripheral nerve features of neuromyotonia (*P* = 0.0003) and neuropathic pain (*P* < 0.0001) were preferentially associated. As expected, the nine patients with antibodies to intracellular VGKC epitopes had heterogeneous, often non-immune, clinical syndromes. By contrast, likely non-immune syndromes were noted in only one patient with LGI1 antibodies (stroke) and in four with CASPR2 antibodies (axonal neuropathy, cervical dystonia, hemifacial spasm and psychosis).

Of greater relevance to a HLA study, patients with LGI1 or CASPR2 antibodies often had co-existent autoimmune conditions (28% and 23%, respectively), including Hashimoto’s thyroiditis (*n* = 8), psoriasis (*n* = 7) and pernicious anaemia (*n* = 2). Moreover, the LGI1-antibody cohort was distinctive for a 47% rate of adverse drug reactions from corticosteroids (*P* = 0.004; 16% with CASPR2 antibodies) and a significantly higher rate of drug-induced rashes (35% versus 3% in CASPR2, *P* = 0.0004). The reported rashes were secondary to AEDs [*n* = 13: including carbamazepine (*n* = 6), phenytoin (*n* = 4), lamotrigine (*n* = 2) and valproate (*n* = 1)], antibiotics [*n* = 6: penicillins (*n* = 5) and metronidazole (*n* = 1)] and immunosuppressants [*n* = 5: azathioprine (*n* = 2), corticosteroids (*n* = 2) and methotrexate (*n* = 1)]. Thus, the LGI1- and CASPR2-antibody groups displayed differing clinical autoimmune features suggesting divergent immunogenetic pathways.

### Patients with LGI1 or CASPR2 antibodies have strong and distinct HLA allelic profiles

**Figure 1:**
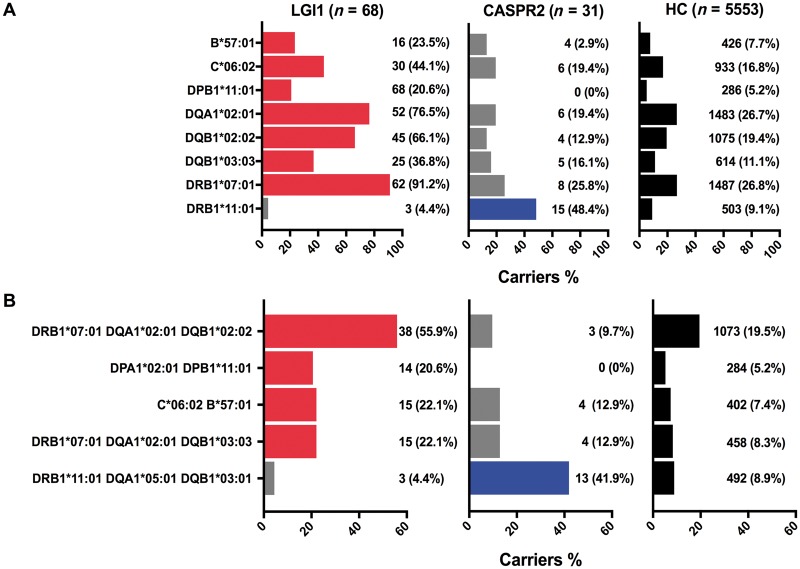
**HLA allele and haplotype associations in patients with LGI1 and CASPR2 antibodies.** Bar chart depicting allele (**A**) and haplotype (**B**) associations and their frequency in patients with antibodies to LGI1 (*n* = 68, red denotes significant associations) and CASPR2 (*n* = 31, blue denotes significant associations), together with the frequency of these alleles or haplotypes in 5553 healthy controls (black bars). HC = healthy controls.

We proceeded to analyse HLA associations, as summarized in Fig. 1 and Table 2. Consistent with previous smaller reports (Kim *et al.*, 2017; van Sonderen *et al.*, 2017), almost all LGI1-antibody-positive patients carried HLA-DRB1*07:01 (91%, compared to 26% in healthy controls) [OR 27.6 (95% confidence interval, CI 12.9–72.2), *P* = 4.1 × 10^−26^]. Further, 13% (9/68) were homozygous for DRB1*07:01, compared to 2% (115/5553) healthy controls [OR 7.3 (95% CI 3.3–14.4), *P* = 3 × 10^−4^]. Alleles recognized to be part of haplotypes involving HLA-DRB1*07:01 (González-Galarza *et al.*, 2015) were over-represented, namely HLA-DQA1*02:01, HLA-DQB1*02:02, HLA-DQB1*03:03 and HLA-DPB1*11:01. Additionally, associations were found with two HLA class I alleles, HLA-B*57:01 [OR = 3.7 (95% CI 2.0–6.5); *P* = 0.014] and HLA-C*06:02 [OR = 3.9 (95% CI 2.4–6.3); *P* = 4.6 × 10^−5^]. After conditioning on the commonest allele, HLA-DRB1*07:01, two other DQ alleles reached statistical significance consistent with evidence of an independent association, HLA-DQA1*01:03 [OR = 4.4 (95% CI 2.2–8.1); *P* = 4 × 10^−3^] and HLA-DRB1*01:03 [OR = 14.7 (95% CI 3.6–51.5), *P* = 0.04].

In striking contrast, analysis of the CASPR2-antibody group identified a single risk allele; HLA-DRB1*11:01, which was present in 48% of CASPR2-antibody patients compared to 4% of patients with LGI1 antibodies and 9% of healthy controls [OR 9.4 (95% CI 4.6–19.3); *P* = 5.7 × 10^−6^]. One CASPR2-antibody patient was homozygous for HLA-DRB1*11:01. Interestingly, the four patients with non-immune conditions and CASPR2 antibodies (Table 1) did not carry HLA-DRB1*11:01, giving it a 56% (15/27) frequency in the remainder. No additional alleles were observed after conditioning on HLA-DRB1*11:01.

Intriguingly, of the three patients with co-existent CASPR2 and LGI1 antibodies, only one carried HLA-DRB1*07:01 and none carried HLA-DRB1*11:01. However, all three carried HLA-B*44:02, HLA-C*05:01, HLA-DQA1*03:01 and HLA-DQB1*03:01, a different complement of alleles to the patients with antibodies to either LGI1 or CASPR2 (Supplementary Fig. 1). There were no significant findings within the group with intracellular VGKC antibodies (Supplementary Fig. 1).

### Haplotype-specific distinctions between patients with LGI1 and CASPR2 antibodies

Next, to understand the *en bloc* allelic inheritance and *in vivo* relevance of HLA combinations that may present LGI1 and CASPR2 antigens, we explored associations involving HLA haplotypes (Fig. 1B and full analysis in Supplementary Figs 2–4). We noted that HLA-DQA1*02:01, HLA-DQB1*02:02, HLA-DQB1*03:03 and HLA-DPB1*11:01 show evidence of linkage disequilibrium with HLA-DRB1*07:01 (r^2^ values 0.64, 0.49, 0.13, 0.10 and D′ 1, 0.95, 0.8 and 1, respectively) (Supplementary material). This was reflected in the most frequent HLA class II haplotypes found in patients with LGI1 antibodies, namely HLA-DRB1*07:01-DQA1*02:01-DQB1*02:02 [OR = 5.2 (95% CI 3.2–8.6); *P* = 2.3 × 10^−9^], DRB1*07:01-DQA1*02:01-DQB1*03:03 [OR = 3.1 (95% CI 1.7–5.5); *P* = 0.02] and DPA1*02:01-DPB1*11:01 [OR = 4.8 (95% CI 2.5–8.5); *P* = 3.8 × 10^−4^]. In addition, LGI1-antibody status was associated with a HLA class I haplotype, HLA-C*06:02-B*57:01 [OR = 3.6 (95% CI 1.9–6.2); *P* = 8.8 × 10^−3^]. By contrast, only one HLA class II haplotype was associated with CASPR2 antibodies: DRB1*11:01-DQA1*05:01-DQB1*03:01 [OR = 7.4 (95% CI 3.5–15.2), *P* = 5.7 × 10^−5^].

Given these significant and distinct allelic and haplotypic HLA associations, for each serologically-defined group, we evaluated their value in explaining sub-phenotypes (limbic encephalitis or epilepsy; peripheral or CNS), long-term outcomes or adverse drug reactions (Supplementary Table 1), and found no significant HLA allele or haplotype associations. However, within LGI1-antibody patients, five of six patients with antibiotic-induced rashes carried HLA-B*57:01 known to associate with risk of rash to abacavir and flucloxacillin (Yip *et al.*, 2015), and four of six patients with psoriasis harboured the psoriasis risk allele C*06:02 (Arakawa *et al.*, 2015), suggesting the extended haplotypes may explain these specific co-morbidities. Finally, from the nine LGI1- and four CASPR2-antibody patients with a tumour, there were no significant HLA differences compared to non-tumour patients (Supplementary Table 2).

### DRB4 analysis

To extend a previous report (van Sonderen *et al.*, 2017), the *HLA-DRB1* paralogue *HLA-DRB4* was sequenced and observed to be absent or a null allele in 61% (19/31) of the CASPR2-antibody cohort and 44% (4/9) of the intracellular VGKC-antibody cohort, consistent with the healthy control frequencies. However, only 16% (11/68) of the LGI1-antibody cohort showed an absent or null HLA-DRB4 allele, and these 11 patients all carried HLA-DRB1*07:01 (Supplementary Table 3).

### Predictions of HLA-binding peptides

**Figure 2:**
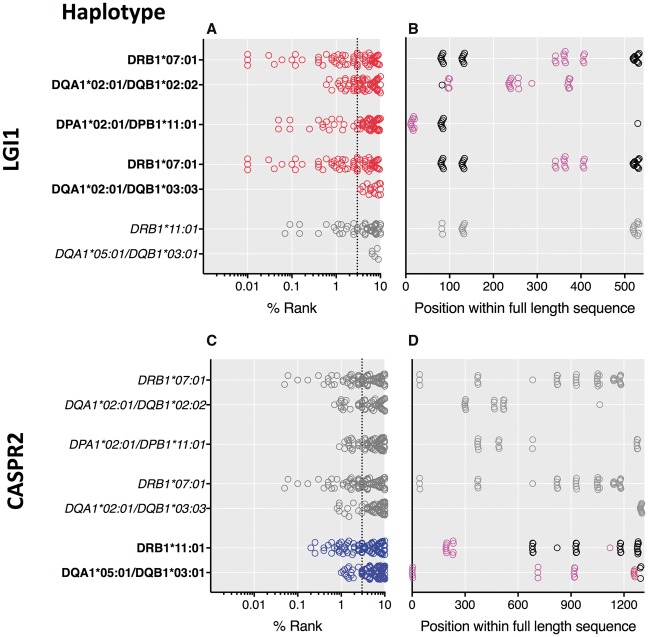
**Peptides derived from full-length LGI1 and CASPR2 predicted to bind MHC-dimers encoded by over-represented HLA haplotypes.** Rankings and position of peptides derived from full-length sequences of LGI1 (**A** and **B**) and CASPR2 (**C** and **D**). The haplotypes correspond to Fig. 1B and when in bold they relate to those observed in patients with antibodies to the corresponding protein. Red circles denote the LGI1-antibody cohort and blue the CASPR2-antibody cohort. Grey circles and italicized haplotypes relate to peptides from the other antigenic protein (i.e. CASPR2 in **A** and **B**; and LGI1 in **C** and **D**). Rank describes the predicted peptide affinities (IC_50_, nM) by comparison to 200 000 random peptides of the same length. Dotted lines represent the 3% cut-off for peptide rank. Within **B** and **D**, circles represent the highly-ranked peptides across the full-length sequences of LGI1 or CASPR2: black circles represent peptides with some predicted promiscuity across LGI1- and CASPR2-antibody HLA variants, whereas pink circles highlight peptides that are not predicted to cross-react.

These robust HLA class II associations strongly implicate CD4^+^ T cells in the pathogenesis of both LGI1- and CASPR2-antibody-associated diseases. To locate potentially high-affinity peptides that complex with HLA class II heterodimers, and may interact with patient T cells, *in silico* modelling was used and focused on all the class II haplotypes identified above (Fig. 2).

Overall, many peptides from both LGI1 and CASPR2 ranked highly for potential binding to several HLA-DR, HLA-DP and HLA-DQ variants (Fig. 2A and C), likely consistent with the varied intrinsic properties of different HLA molecules. Furthermore, for HLA-DRB1*07:01 and HLA-DRB1*11:01, which pair with the invariant DRA chain, and for HLA-DQA1*02:01-DQB1*02:02 heterodimers, peptide ranks showed little difference between LGI1- and CASPR2-derived peptides, suggesting a lack of antigen selectivity. Also, no highly-ranked peptides were identified to bind the LGI1-antibody-associated heterodimer HLA-DQA1*02:01-DQB1*03:03, making it an unlikely candidate molecule for LGI1 peptide presentation. By contrast, the CASPR2-antibody-associated HLA-DQA1*05:01-DQB1*03:01 heterodimer was predicted to bind some high-ranking peptides from the CASPR2 sequence only, suggesting CASPR2 specificity.

As expected for the shared core sequences between consecutive 15-mers, many highly ranked peptides were from tightly clustered locations within the full-length protein (Fig. 2B, D and Supplementary Table 4). Most peptides within these clusters showed potential to bind the HLA variants observed in both the LGI1- and CASPR2-antibody cohorts (Fig. 2B and D, black circles). This included one previously identified peptide (Kim *et al.*, 2017) and argues against its role in disease specificity. However, 9/13 LGI1-derived peptides and 7/13 from the CASPR2 sequence showed binding potential that was more restricted to the variants associated with the corresponding antibody cohort (Fig. 2B and D, pink circles). From LGI1, 4/9 core peptides were predicted to bind with high affinity (<40 nM), typically to HLA-DRB1*07:01, although interestingly the highest affinity peptide was predicted to bind HLA-DPA1*02:01-DPB1*11:01 (Supplementary Table 4). From CASPR2-derived peptides, 7/7 were predicted to bind with high affinity, distributed across the variants within the CASPR2-antibody-associated haplotype (Supplementary Table 4).

## Discussion

This study is the first comparative HLA analysis of LGI1- and CASPR2-autoantibody mediated diseases, and shows marked and strikingly different HLA associations for these patients, at both allelic and haplotypic levels. Given the frequently overlapping clinical features in patients with LGI1 and CASPR2 antibodies, and their co-expression in VGKC complexes, these findings indicate that dichotomous predisposing HLA variants govern the generation of LGI1 versus CASPR2 antibodies. Furthermore, they strongly implicate T cells in disease initiation and the candidate HLA-binding peptide partners generated by our *in silico* data may help identify these interacting T cells.

While HLA-DRB1*07:01 and linked class II alleles, including the haplotype HLA-DRB1*07:01-DQA1*02:01-DQB1*02:02, showed very strong associations with LGI1-antibody patients, this was not observed among CASPR2-antibody patients in whom we found clear associations with HLA-DRB1*11:01 only. Among LGI1-antibody patients, DRB1*11:01 was observed at around healthy control rates, DRB4 was less frequently detected than DRB1*07:01, homozygosity for HLA-DRB1*07:01 was recognized, and other independent associations involved HLA class I alleles HLA-B*57:01 and HLA-C*06:02. Albeit limited by their intrinsic rarity, intriguingly, the three patients with both LGI1 and CASPR2 antibodies had yet another complement of HLA alleles. Perhaps this implicates further divergence in molecular mechanisms responsible for the generation of both autoantibody specificities within an individual. However, the HLA associations do not appear to distinguish between sub-phenotypes, outcomes or, in contrast to a previous observation, the presence of associated tumours (van Sonderen *et al.*, 2017). Also, the 9–27% frequencies of these HLA variants in healthy Caucasians are far higher than disease prevalence, implicating additional loci, environmental or stochastic influences in disease manifestation.

Furthermore, our data also provide several intriguing insights into the immunopathogenesis of these diseases. First, they extend the frequent HLA associations in IgG4-related diseases (Huijbers *et al.*, 2015), but here, exceptionally, with no DQ5 association. Second, the presence of dominant HLA class II associations implicates extracellular antigen processing and CD4 T cells in disease initiation (Trowsdale and Knight, 2013), but the LGI1 antibody class I associations found here, and HLA-B*44:03 and HLA-C*07:06 reported in seven patients previously (Kim *et al.*, 2017), are compatible with a role for intracellular antigen processing, including viruses and drugs. These class I differences between studies may be explained by ethnicity, sample size and relatively weak associations (Kim *et al.*, 2017; van Sonderen *et al.*, 2017). Indeed, this extended haplotype and the related complex linkage disequilibrium in this region of the genome warrant further analysis. Furthermore, our original hypothesis of adverse drug reaction-related HLA variants may relate to the linked adverse drug reaction-related class I and II HLA variants (HLA-DRB1*07:01, HLA-DQA1*02:01, HLA-B*57:01) (Yip *et al.*, 2015). These and future observations in patients with LGI1 antibodies may inform the genetic basis of more common adverse drug reactions. Third, the HLA similarities between tumour and non-tumour LGI1-antibody cases suggests the absence of a unique paraneoplastic signature, in contrast to Lambert-Eaton myasthenic syndrome (Wirtz *et al.*, 2005). Perhaps this implies tumours in patients with LGI1 antibodies largely reflect the age-matched background rate, rather than a distinct immune mechanism, although a paucity of tumours classically associated with paraneoplastic neurological syndromes may limit our interpretation. Finally, our *in silico* predictions suggest that HLA-DQA1*02:01-DQB1*03:03 is unlikely to mediate presentation of LGI1-derived peptides, whereas the HLA-DQA1*05:01-DQB1*03:01 heterodimer may be implicated in the CASPR2-antibody phenotype. Also, the promiscuity of both CASPR2 and LGI1 peptides for some HLA variants, including HLA-DRB1*07:01 (Kim *et al.*, 2017), may explain why immunization with the same VGKC complexes may generate two distinct disease entities, and underlie the observed co-existence of both antibodies at rates far higher than expected by chance (Irani *et al.*, 2012). However, this *in silico* approach is inherently limited by the possibility that high affinity peptides are more effectively deleted through central tolerance. Nevertheless, taken together, the range of antigen-restricted peptides derived herein, and the relative HLA variant frequencies in disease versus control populations, generate hypothesis-driven approaches to expand disease-specific T cells *in vitro* and complement recent clinical and laboratory observations which strongly implicate T cell dependence of antibody-mediated diseases (Makuch *et al.*, 2018; Wilson *et al.*, 2018*a*, *b*).

In summary, the distinct HLA associations in patients with LGI1 and CASPR2 autoantibodies, together with differing clinical features relating to autoimmunity, support an immunological dissociation in generation of these clinically-overlapping autoantibody-mediated syndromes. The dominant class II HLA involvement combined with *in silico* predictions, offers potential to better understand the likely initiating T–B cell interactions. Further work should focus on the environmental factors that influence the presentation of peptides in genetically predisposed individuals.

**Table 2:** Significant allele (*top*) and haplotype (*bottom*) associations in patients with LGI1 (*n* = 68) or CASPR2 (*n* = 31) antibodies

Antibody group	Allele / haplotype	OR	95% CI lower	95% CI upper	Fisher’s exact test	Corrected (Hochberg) *P*-value
LGI1	B*57:01	3.7	2.0	6.5	5.2 × 10^−5^	1.0 × 10^−2^
LGI1	C*06:02	3.9	2.4	6.3	1.6 × 10^−7^	4.6 × 10^−5^
LGI1	DPB1*11:01	4.9	2.6	8.7	8.4 × 10^−6^	2.0 × 10^−3^
LGI1	DQA1*02:01	8.9	5.2	16.1	3.0 × 10^−17^	8.7 × 10^−15^
LGI1	DQB1*02:02	8.1	4.9	13.7	9.5 × 10^−17^	2.8 × 10^−14^
LGI1	DQB1*03:03	4.7	2.8	7.7	3.6 × 10^−8^	1.0 × 10^−5^
LGI1	DRB1*07:01	27.6	12.9	72.2	1.4 × 10^−28^	4.1 × 10^−26^
CASPR2	DRB1*11:01	9.4	4.6	19.3	2.0 × 10^−8^	5.7 × 10^−6^

LGI1	DRB1*07:01-DQA1*02:01-DQB1*02:02	5.2	3.2	8.6	4.7 × 10^−11^	2.3 × 10^−9^
LGI1	DRB1*07:01-DQA1*02:01-DQB1*03:03	3.1	1.7	5.5	4.4 × 10^−4^	2.1 × 10^−2^
LGI1	DPA1*02:01-DPB1*11:01	4.8	2.5	8.5	1.0 × 10^−5^	3.8 × 10^−4^
LGI1	C*06:02-B*57:01	3.6	1.9	6.2	1.3 × 10^−4^	8.8 × 10^−3^
CASPR2	DRB1*11:01-DQA1*05:01-DQB1*03:01	7.4	3.5	15.2	1.1 × 10^−6^	5.7 × 10^−5^

Corrected *P-*values indicate comparison between disease and healthy controls.

## Supplementary Material

Supplementary Data

## Abbreviations

HLA: human leucocyte antigen
VGKC: voltage-gated potassium channel

## References

[awy109-B1] Andreatta M, Karosiene E, Rasmussen M, Stryhn A, Buus S, Nielsen M Accurate pan-specific prediction of peptide-MHC class II binding affinity with improved binding core identification. Immunogenetics 2015; 67: 641–50. 26416257 10.1007/s00251-015-0873-y PMC4637192

[awy109-B2] Arakawa A, Siewert K, Stöhr J, Besgen P, Kim SM, Rühl G et al Melanocyte antigen triggers autoimmunity in human psoriasis. J Exp Med 2015; 212: 2203–12. 26621454 10.1084/jem.20151093 PMC4689169

[awy109-B3] Ariño H, Armangue T, Petit-Pedrol M, Sabater L, Martinez-Hernandez E, Hara M et al Anti-LGI1-associated cognitive impairment: presentation and long-term outcome. Neurology 2016; 87: 759–65. 27466467 10.1212/WNL.0000000000003009 PMC4999321

[awy109-B4] Binks SNM, Klein CJ, Waters P, Pittock SJ, Irani SR LGI1, CASPR2 and related antibodies: a molecular evolution of the phenotypes. J Neurol Neurosurg Psychiatry 2018; 89: 526–34. 29055902 10.1136/jnnp-2017-315720 PMC5909759

[awy109-B5] Bunce M, O'Neill CM, Barnardo MC, Krausa P, Browning MJ, Morris PJ et al Phototyping: comprehensive DNA typing for HLA-A, B, C, DRB1, DRB3, DRB4, DRB5 & DQB1 by PCR with 144 primer mixes utilizing sequence-specific primers (PCR-SSP). Tissue Antigens 1995; 46: 355–67. 8838344 10.1111/j.1399-0039.1995.tb03127.x

[awy109-B6] Gadoth A, Pittock SJ, Dubey D, McKeon A, Britton JW, Schmeling JE et al Expanded phenotypes and outcomes among 256 LGI1/CASPR2-IgG positive patients. Ann Neurol 2017; 82: 79–92. 28628235 10.1002/ana.24979

[awy109-B7] González-Galarza FF, Takeshita LY, Santos EJ, Kempson F, Maia MH, da Silva AL et al Allele frequency net 2015 update: new features for HLA epitopes, KIR and disease and HLA adverse drug reaction associations. Nucleic Acids Res 2015; 43: D784–8. 25414323 10.1093/nar/gku1166 PMC4383964

[awy109-B8] Graus F, Gorman MP Voltage-gated potassium channel antibodies: game over. Neurology 2016; 86: 1657–8. 27037235 10.1212/WNL.0000000000002644

[awy109-B9] Huijbers MG, Querol LA, Niks EH, Plomp JJ, van der Maarel SM, Graus F et al The expanding field of IgG4-mediated neurological autoimmune disorders. Eur J Neurol 2015; 22: 1151–61. 26032110 10.1111/ene.12758

[awy109-B10] Irani SR, Alexander S, Waters P, Kleopa KA, Pettingill P, Zuliani L et al Antibodies to Kv1 potassium channel-complex proteins leucine-rich, glioma inactivated 1 protein and contactin-associated protein-2 in limbic encephalitis, Morvan's syndrome and acquired neuromyotonia. Brain 2010; 133: 2734–48. 20663977 10.1093/brain/awq213 PMC2929337

[awy109-B11] Irani SR, Michell AW, Lang B, Pettingill P, Waters P, Johnson MR et al Faciobrachial dystonic seizures precede Lgi1 antibody limbic encephalitis. Ann Neurol 2011; 69: 892–900. 21416487 10.1002/ana.22307

[awy109-B12] Irani SR, Pettingill P, Kleopa KA, Schiza N, Waters P, Mazia C et al Morvan syndrome: clinical and serological observations in 29 cases. Ann Neurol 2012; 72: 241–55. 22473710 10.1002/ana.23577

[awy109-B13] Irani SR, Stagg CJ, Schott JM, Rosenthal CR, Schneider SA, Pettingill P et al Faciobrachial dystonic seizures: the influence of immunotherapy on seizure control and prevention of cognitive impairment in a broadening phenotype. Brain 2013; 136: 3151–62. 24014519 10.1093/brain/awt212

[awy109-B14] Jia X, Han B, Onengut-Gumuscu S, Chen WM, Concannon PJ, Rich SS et al Imputing amino acid polymorphisms in human leukocyte antigens. PLoS One 2013; 8: e64683. 23762245 10.1371/journal.pone.0064683 PMC3675122

[awy109-B16] Kim TJ, Lee ST, Moon J, Sunwoo JS, Byun JI, Lim JA et al Anti-LGI1 encephalitis is associated with unique HLA subtypes. Ann Neurol 2017; 81: 183–92. 28026029 10.1002/ana.24860

[awy109-B17] Klein CJ, Lennon VA, Aston PA, McKeon A, O’Toole O, Quek A et al Insights from LGI1 and CASPR2 potassium channel complex autoantibody subtyping. JAMA Neurol 2013; 70: 229–34. 23407760 10.1001/jamaneurol.2013.592 PMC3895328

[awy109-B18] Lai M, Huijbers MG, Lancaster E, Graus F, Bataller L, Balice-Gordon R et al Investigation of LGI1 as the antigen in limbic encephalitis previously attributed to potassium channels: a case series. Lancet Neurol 2010; 9: 776–85. 20580615 10.1016/S1474-4422(10)70137-X PMC3086669

[awy109-B19] Lang B, Makuch M, Moloney T, Dettmann I, Mindorf S, Probst C et al Intracellular and non-neuronal targets of voltage-gated potassium channel complex antibodies. J Neurol Neurosurg Psychiatry 2017; 88: 353–61. 28115470 10.1136/jnnp-2016-314758 PMC5644714

[awy109-B34] Makuch M, Wilson R, Al-Diwani A, Varley J, Kienzler AK, Taylor J et al N-methyl-D-aspartate receptor antibody production from germinal center reactions: therapeutic implications. Ann Neurol 2018; 83: 553–61. 29406578 10.1002/ana.25173 PMC5925521

[awy109-B151] McCormack M, Alfirevic A, Bourgeois S, Farrell JJ, Kasperavičiūtė D, Carrington M et al HLA-A*3101 and carbamazepine-induced hypersensitivity reactions in Europeans. N Engl J Med 2011; 364: 1134–43. 21428769 10.1056/NEJMoa1013297 PMC3113609

[awy109-B20] Meeusen JW, Klein CJ, Pirko I, Haselkorn KE, Kryzer TJ, Pittock SJ et al Potassium channel complex autoimmunity induced by inhaled brain tissue aerosol. Ann Neurol 2012; 71: 417–26. 22451206 10.1002/ana.22674 PMC3315155

[awy109-B21] Neville MJ, Lee W, Humburg P, Wong D, Barnardo M, Karpe F et al High resolution HLA haplotyping by imputation for a British population bioresource. Hum Immunol 2017; 78: 242–51. 28111166 10.1016/j.humimm.2017.01.006 PMC5367449

[awy109-B23] Stephens M, Donnelly P A comparison of bayesian methods for haplotype reconstruction from population genotype data. Am J Hum Genet 2003; 73: 1162–9. 14574645 10.1086/379378 PMC1180495

[awy109-B24] Stephens M, Smith NJ, Donnelly P A new statistical method for haplotype reconstruction from population data. Am J Hum Genet 2001; 68: 978–89. 11254454 10.1086/319501 PMC1275651

[awy109-B25] Thieben MJ, Lennon VA, Boeve BF, Aksamit AJ, Keegan M, Vernino S Potentially reversible autoimmune limbic encephalitis with neuronal potassium channel antibody. Neurology 2004; 62: 1177–82. 15079019 10.1212/01.wnl.0000122648.19196.02

[awy109-B26] Thompson J, Bi M, Murchison AG, Makuch M, Bien CG, Chu K et al The importance of early immunotherapy in patients with faciobrachial dystonic seizures. Brain 2018; 141: 348–56. 29272336 10.1093/brain/awx323 PMC5837230

[awy109-B27] Trowsdale J, Knight JC Major histocompatibility complex genomics and human disease. Annu Rev Genom Hum Genet 2013; 14: 301–23. 10.1146/annurev-genom-091212-153455 PMC4426292 23875801

[awy109-B28] van Sonderen A, Roelen DL, Stoop JA, Verdijk RM, Haasnoot GW, Thijs RD et al Anti-LGI1 encephalitis is strongly associated with HLA-DR7 and HLA-DRB4. Ann Neurol 2017; 81: 193–8. 28026046 10.1002/ana.24858

[awy109-B29] van Sonderen A, Schreurs MW, de Bruijn MA, Boukhrissi S, Nagtzaam MM, Hulsenboom ES et al The relevance of VGKC positivity in the absence of LGI1 and Caspr2 antibodies. Neurology 2016; 86: 1692–9. 27037230 10.1212/WNL.0000000000002637

[awy109-B30] Vincent A, Buckley C, Schott JM, Baker I, Dewar BK, Detert N et al Potassium channel antibody-associated encephalopathy: a potentially immunotherapy-responsive form of limbic encephalitis. Brain 2004; 127: 701–12. 14960497 10.1093/brain/awh077

[awy109-B35] Wilson R, Makuch M, Kienzler AK, Varley J, Taylor J, Woodhall M et al Condition-dependent generation of aquaporin-4 antibodies from circulating B cells in Neuromyelitis Optica. Brain 2018a; 141: 1063–74. 29447335 10.1093/brain/awy010 PMC5889028

[awy109-B36] Wilson R, Menassa DA, Davies AJ, Michael S, Hester JO, Kuker W et al Seronegative antibody-mediated neurology after immune checkpoint inhibitors. Ann Clin Transl Neurol 2018b; 17: 305–6. 10.1002/acn3.547 PMC5945956 29761126

[awy109-B32] Wirtz PW, Willcox N, van der Slik AR, Lang B, Maddison P, Koeleman BP et al HLA and smoking in prediction and prognosis of small cell lung cancer in autoimmune Lambert-Eaton myasthenic syndrome. J Neuroimmunol 2005; 159: 230–7. 15652424 10.1016/j.jneuroim.2004.10.018

[awy109-B33] Yip VL, Alfirevic A, Pirmohamed M Genetics of immune-mediated adverse drug reactions: a comprehensive and clinical review. Clin Rev Allergy Immunol 2015; 48: 165–75. 24777842 10.1007/s12016-014-8418-y

