# Supplementary material for: Distinct HLA associations of LGI1 and CASPR2-antibody diseases
Source: Brain. 2018 May 18;141(8):2263–71. doi: 10.1093/brain/awy109 (PMC6118231; doi:10.1093/brain/awy109)
Supplement: Supplementary Data [file awy109_brain-2017-01695-File004.pdf]

Supplementary Methods

Genotyping, data processing and quality control

Genomic DNA was extracted from whole blood using the Qiagen DNeasy extraction kit (Qiagen, Manchester UK) and quality assessed using 260/280nm spectrophotometer ratios (LGC Genomics, Hoddesdon, UK). Samples were genotyped using the Illumina Infinium Global Screening Array-24+v1.0 beadchip array (Illumina, San Diego, CA). Variants were called using the Illumina GenCall algorithm (Teo *et al.*, 2007). Samples were excluded based on a call rate <98%, heterozygosity 3SD of mean and exact Hardy-Weinberg equilibrium (HWE) $<10^{-4}$.

Imputation and HLA typing

Imputation of HLA alleles was performed using SNP2HLA software, as described previously (Jia *et al.*, 2013), to impute two- and four-digit resolution classical HLA alleles for the classical HLA-A, -C, -B, -DRB1, -DQA1, -DQB1, -DPA1 and -DPB1 gene loci. In brief, SNP2HLA_package_v1.0.2, Beagle.3.0.4, linkage2beagle_2.0 and Plink1.07 were used following recommended parameters (10 iterations and maximum window size of 1000 bp) as described (Neville *et al.*, 2017). The pre-built Type 1 Diabetes Genetics Consortium (T1DGC) Immunochip/HLA reference panel of 8,961 binary markers from 5,225 unrelated individuals with European ancestry was used as training set for the HLA imputation (Jia *et al.*, 2013). There was an overlap of 4,708 SNP markers between the case dataset and the reference dataset. Thresholds of 0.8 and 0.3 for posterior probability and genotype correlation respectively were applied for data quality control. In broad agreement with published data, DRB1- and DQ-alleles were not determined by imputation in 49/222 (22%) and

30/444 (7%) of alleles, respectively (Neville *et al.*, 2017). SNP2HLA demonstrated high accuracy with 95% (210/222) calls for DRB1 alleles unaltered after tissue typing. PCR-SSP data were confirmed by Sanger sequencing-based typing (PCR-SBT) to six digit resolution (Blasczyk *et al.*, 1998) for the most frequent allele, DRB1*07:01. Linkage disequilibrium was evaluated using r2 and D’ values (Wang *et al.*, 2005). HLA-DRB4 was typed at intermediate resolution in all patients to establish DRB4 carriers, and frequencies of null and absent alleles.

References

Blasczyk R, Kotsch K, Wehling J. The nature of polymorphism of the HLA-DRB intron sequences is lineage specific. Tissue Antigens 1998; 52: 19–26.

Jia X, Han B, Onengut-Gumuscu S, Chen W-M, Concannon PJ, Rich SS, et al. Imputing amino acid polymorphisms in human leukocyte antigens. PLoS ONE 2013; 8: e64683.

Neville MJ, Lee W, Humburg P, Wong D, Barnardo M, Karpe F, et al. High resolution HLA haplotyping by imputation for a British population bioresource. Hum. Immunol. 2017; 78: 242–251.

Teo YY, Inouye M, Small KS, Gwilliam R, Deloukas P, Kwiatkowski DP, et al. A genotype calling algorithm for the Illumina BeadArray platform. Bioinformatics 2007; 23: 2741–2746.

Wang WYS, Barratt BJ, Clayton DG, Todd JA. Genome-wide association studies: theoretical and practical concerns. Nat Rev Genet 2005; 6: 109–118.

Supplementary Table 1. Endophenotypic analyses performed within the LGI1 and CASPR2 cohorts

**LGI1-antibody patients: allele analyses**
Presentation with limbic encephalitis, epilepsy or neuromyotonia
Modified Rankin Score (mRS) change ≤1 or ≥2
Drug rash present or absent
Adverse effect of steroids present or absent
Tumour present or absent
**CASPR2-antibody patients: allele analyses**
Presentation with limbic encephalitis, epilepsy or neuromyotonia
Modified Rankin Score (mRS) change ≤1 or ≥2
Neuropathic pain present or absent
Tumour present or absent
**LGI1-antibody patients: haplotype analyses**
Drug rash present or absent
Adverse effect of steroids present or absent

Supplementary Table 2. DQA1-, DQB1-, and DRB1-alleles of 13 patients with tumours. Nine patients had LGI1-antibodies and four CASPR2-antibodies.

**LGI1-antibody with tumour (n=9)**					
**Age, sex**	**Syndrome**	**DQA1**	**DQB1**	**DRB1**	**Tumour type**
79, F	Epilepsy	*05:01/ *02:01	*02:01/ *02:02	*07:01/ *03:01	Basal cell carcinoma (BCC)
51, M	LE	*01:01/ *02:01	*05:01/ *03:03	*07:01/ *01:03	BCC
67, M	LE	*03:01/ *02:01	*03:03/ *02:02	*07:01/ *09:01	Bladder
70, M	Epilepsy	*02:01/ *02:01	*02:02/ *02:02	*07:01/ *07:01	Skin, type not known
64, F	LE	*03:01/ *02:01	*03:03/ *02:02	*07:01/ *09:01	Breast (remote history)
76, M	Epilepsy	*03:01/ *02:01	*03:03/ *02:02	*07:01/ *09:01	Scalp, squamous or BCC
53, M	LE	*02:01/ *02:01	*02:02/ *02:02	*07:01/ *07:01	Adenocarcinoma of prostate
64, M	LE	*03:01/ *02:01	*03:01/ *02:02	*07:01/ *04:01	BCC
63, M	Epilepsy	*01:03/ *01:03	*03:03/ *02:02	*07:01/ *07:01	Dysplastic colonic polyp
**CASPR2-antibody with tumour (n=4)**					
**Age, sex**	**Syndrome**	**DQA1**	**DQB1**	**DRB1**	**Tumour type**
74, M	LE	*02:01/ *05:01	*03:03/ *03:01	*07:01/ *11:02	Thymic cyst
71, M	Epilepsy	*05:01/ *01:01	*03:01/ *05:03	*14:54/ *11:01	Prostate
72, M	Epilepsy	*01:01/ *01:01	*05:01/ *05:03	*14:01/ *01:01	Prostate
74, M	LE	*02:01/ *02:01	*02:02/ *03:03	*07:01/ *07:01	Pancreatic

Supplementary Table 3. Proportion of patients who do not carry DRB4 alleles or express null DRB4 alleles. Patients were divided into three groups: i) DRB4 expressed (individuals carrying at least one copy of a DRB4 expressed allele), ii) DRB4 not carried (individuals carrying no DRB4 alleles) and iii) DRB4 null only (individuals carrying one or two copies of a DRB4 null allele and zero copies of a DRB4 expressed allele). The null *HLA-DRB4* allele is known to be in linkage disequilibrium with the DQB1*07:01-DQB1*03:03 haplotype, whereas *HLA-DRB4* is typically expressed within the DQB1*07:01-DQB1*02:02 haplotype.

Antibody	DRB4 expressed	DRB4 not carried	DRB4 null only
LGI1 (n=68)	57 (83.8%)	2 (2.9%)	9 (13.2%)
CASPR2 (n=31)	12 (38.7%)	16 (51.6%)	3 (9.7%)
CASPR2/LGI1 (n=3)	3 (100%)	0 (0%)	0 (0%)
VGKC (n=9)	5 (55.5%)	4 (44.4%)	0 (0%)
Controls (n=5133)*	2578 (50.2%)	2306 (44.9%)	249 (4.9%)

*Local data based on consecutive deceased organ donors and patients awaiting stem cell transplantation (http://odt.nhs.uk/pdf/antigen.pdf).

	15-mer start position	Extended core	LGI1 allele(s) binding	Highest affinity [nM]	CASPR2 allele(s) binding
**LGI1**	12-21	KRIAYFLCLLSALLL	DPA1*02:01/DPB1*11:01	14.07	-
	81-87	FLFTPSLQLLL	DRB1*07:01; DPA1*02:01/DPB1*11:01; DQA1*02:01/DQB1*02:02	5.79	DRB1*11:01
	97-101	FDVISDDAF	DQA1*02:01/DQB1*02:02	722.35	-
	129-135	FRGLKSLIHLSL	DRB1*07:01	8.82	DRB1*11:01
	236-242	FSYLNDEYVVIAQPF	DQA1*02:01/DQB1*02:02	594.18	-
	257-259	IFLEWDHVE	DQA1*02:01/DQB1*02:02	880.63	-
	289	LYVIVAQLF	DQA1*02:01/DQB1*02:02	924.59	-
	342-344	FVVADSSKA	DRB1*07:01	36.94	-
	362-366	FYSHQSLHA	DRB1*07:01	25.49	-
	371-376	WYRDTDVEY	DQA1*02:01/DQB1*02:02	440.50	-
	406-409	YQWNKATQL	DRB1*07:01	26.51	-
	520-524	FTHVSINKR	DRB1*07:01	22.60	DRB1*11:01
	529-534	FLFASSFKGN	DRB1*07:01; DPA1*02:01/DPB1*11:01	15.77	DRB1*11:01
	**15-mer start position**	**Extended core**	**CASPR2 allele(s) binding**	**Highest affinity [nM]**	**LGI1 allele(s) binding**
**CASPR2**	1-5	PRAGCGAALLL	DQA1*05:01/DQB1*03:01	15.65	-
	192-197	YRFRNKKMK	DRB1*11:01	21.90	-
	232-235	YITLELKKAKLV	DRB1*11:01	27.15	-
	682-687	YFCKMSRLLNT	DRB1*11:01	16.94	DRB1*07:01; DPA1*02:01/DPB1*11:01
	713-715	WGGSGPGIQ	DQA1*05:01/DQB1*03:01	23.53	-
	823	FKTLTPWGV	DRB1*11:01	34.05	DRB1*07:01
	918-923	FVGGAGGQQG	DQA1*05:01/DQB1*03:01	12.95	-

	928-935	FLGCIRSLRMNGV	DRB1*11:01	17.26	DRB1*07:01
	1122	FLKLDHYPS	DRB1*11:01	36.97	-
	1178-1184	FNQIAPLKAALR	DRB1*11:01	13.04	DRB1*07:01
	1257-1262	IIGGVIAVV	DQA1*05:01/DQB1*03:01	13.39	-
	1276-1283	LIRYMFRHKG	DRB1*11:01	10.82	DPA1*02:01/DPB1*11:01
	1293-1298	AKGAESAESADAAIM	DQA1*05:01/DQB1*03:01	23.07	DQA1*02:01/DQB1*03:03

Supplementary Table 4. Peptides derived from LGI1 and CASPR2 sequences and HLA binding partners. The peptides from LGI1 or CASPR2 which are predicted to bind HLA variants derived from the haplotype analyses. The positions of the peptide clusters within the full-length molecule (15-mer starting position), the extended core amino acid sequence, highest affinity of the peptides in the cluster (nM), and predicted binding to LGI1 and/or CASPR2-cohort haplotypes.

Supplementary Figure 1. HLA-allele associations across the VGKC-complex. HLA Class I and II allele frequencies across patients with autoantibodies to LGI1 (n=68), CASPR2 (n=31), intracellular domains of VGKCs (n=9) and both LGI1 and CASPR2 (n=3), and in healthy controls (n=5553). Colours represent adjusted p values of alleles overrepresented in patients. Given very high proportions of patients with some alleles, under-representations have not been considered, as they are likely to reflect this bias rather than protective alleles.

Supplementary Figure 2. HLA DR-DQ haplotype block associations. HLA DR-DQ haplotypes across patients with autoantibodies to LGI1 (n=68), CASPR2 (n=31), intracellular domains of VGKCs (n=9) and both LGI1 and CASPR2 (n=3), and in healthy controls (n=5553). Colours represent adjusted p values of haplotypes overrepresented in patients.

Supplementary Figure 3. HLA DP haplotype block associations. HLA DP haplotype across patients with autoantibodies to LGI1 (n=68), CASPR2 (n=31), intracellular domains of VGKCs (n=9) and both LGI1 and CASPR2 (n=3), and in healthy controls (n=5553). Single colour represents adjusted p value of haplotype overrepresented in LGI1-antibody patients.

Supplementary Figure 4. HLA-B and C haplotype block associations. HLA-B and C haplotypes across patients with autoantibodies to LGI1 (n=68), CASPR2 (n=31), intracellular domains of VGKCs (n=9) and both LGI1 and CASPR2 (n=3),

and in healthy controls (n=5553). Single colour represents adjusted p value of haplotype overrepresented in LGI1-antibody patients.

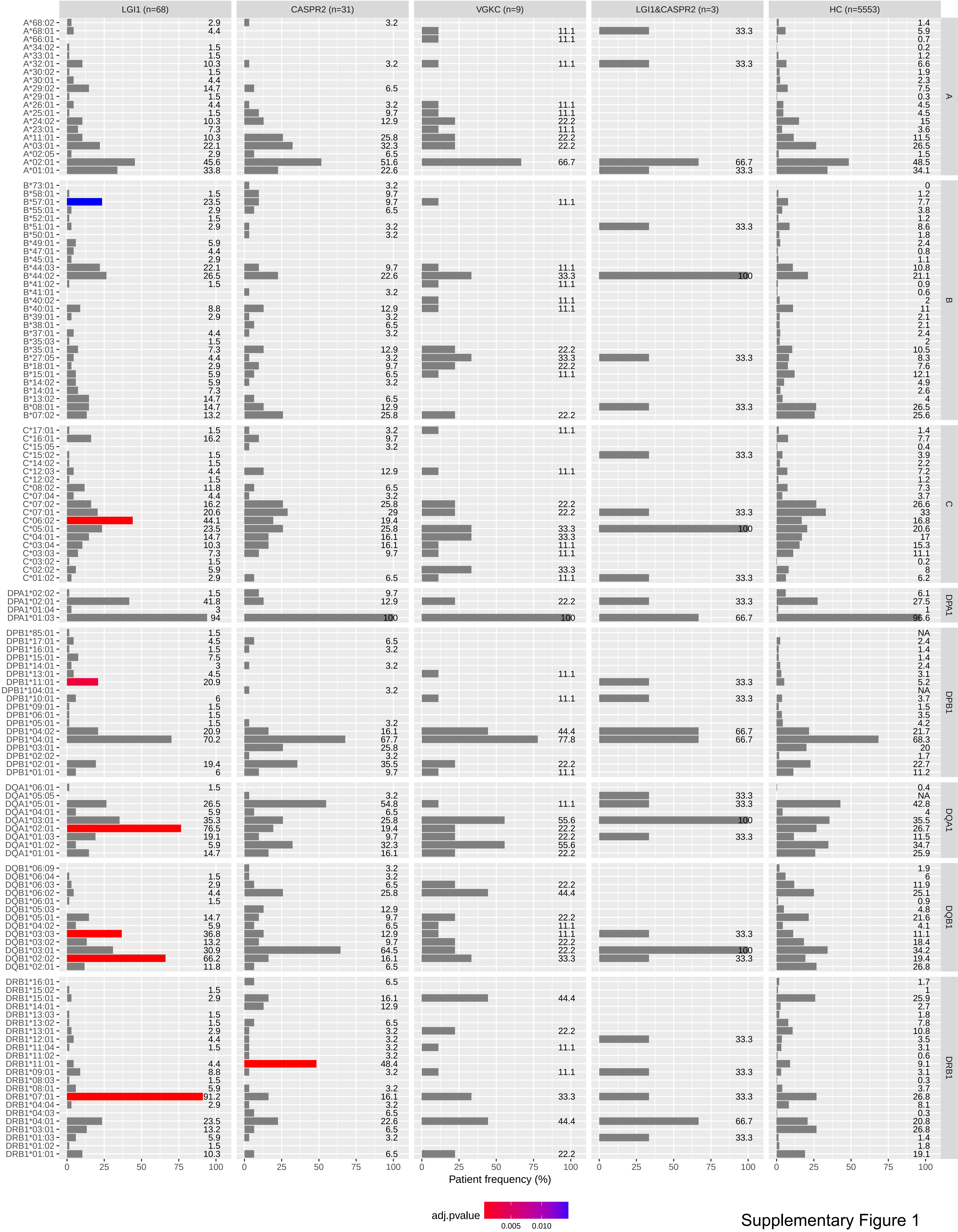

Supplementary Figure 1

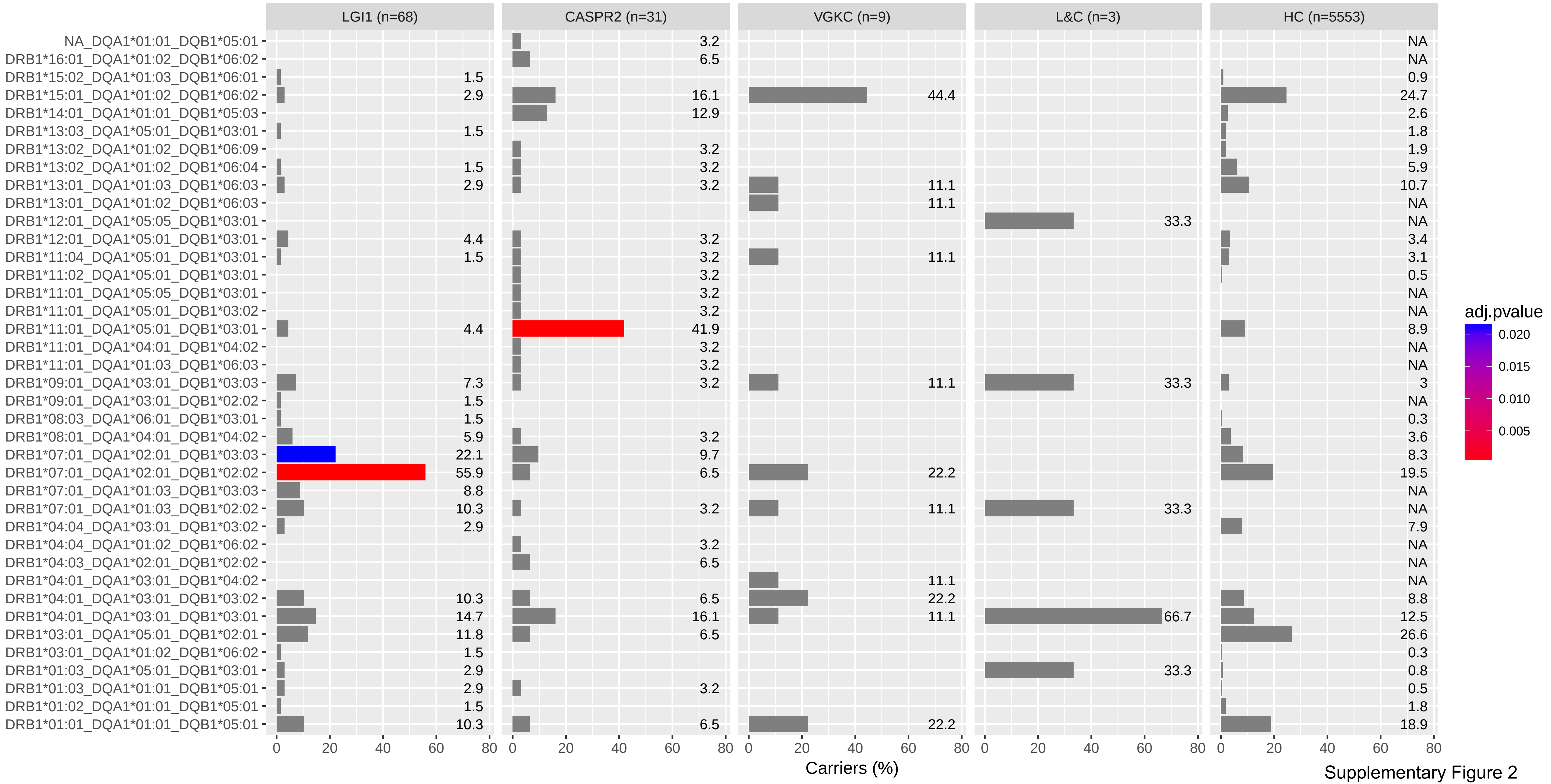

Supplementary Figure 2

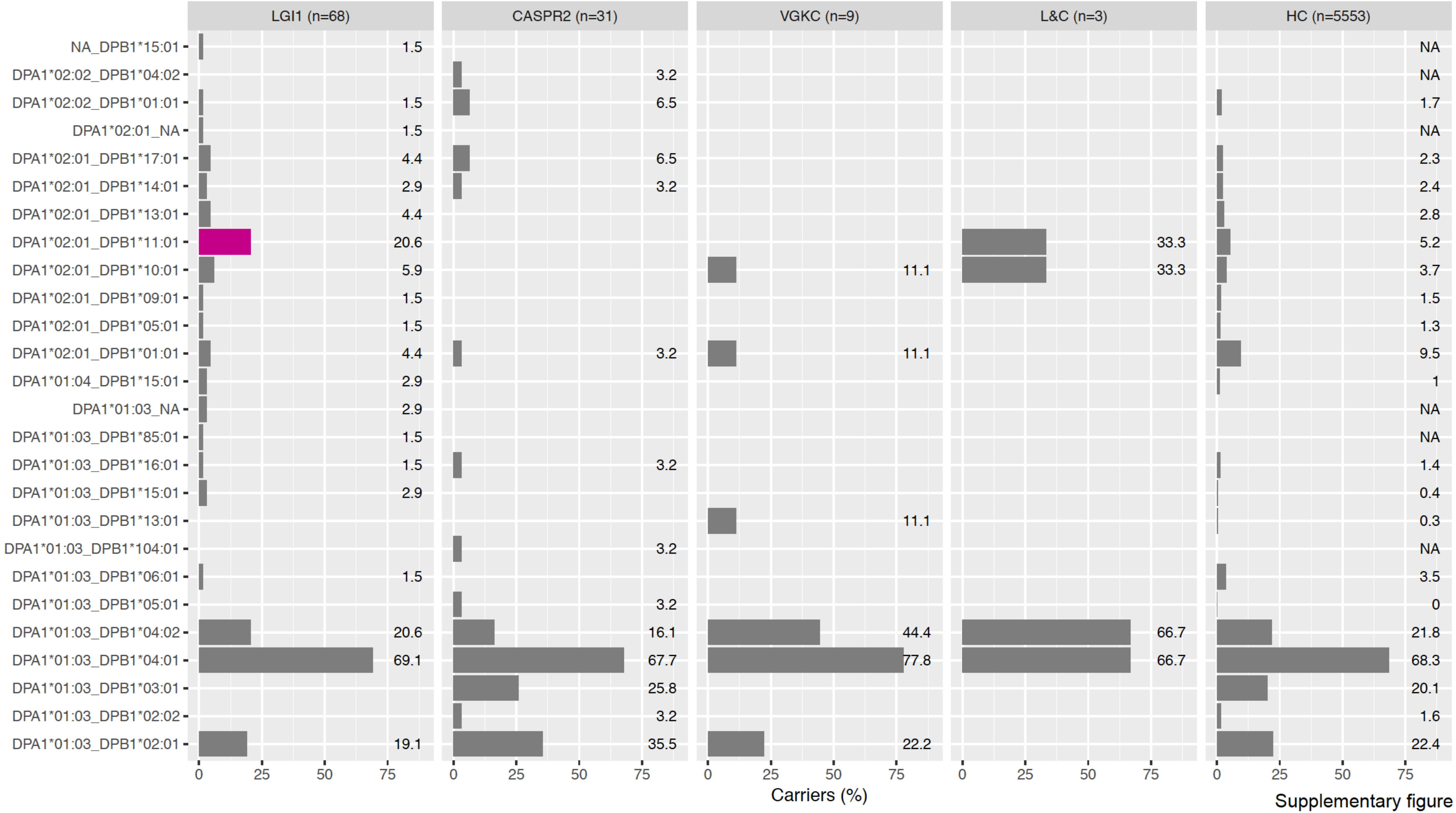

Supplementary figure 3

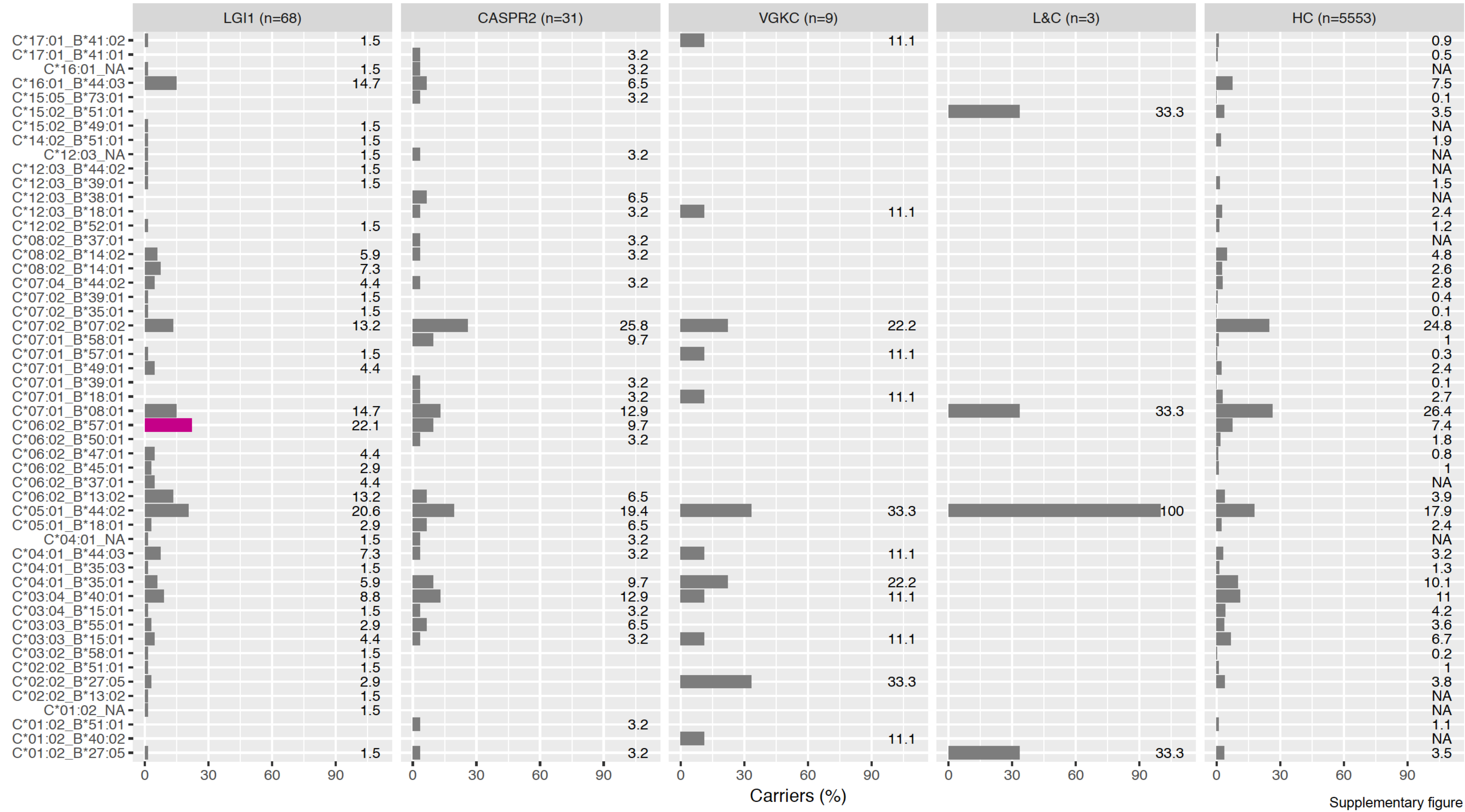

Supplementary figure
